# Supplementary material for: The pupal moulting fluid has evolved social functions in ants
Source: Nature. 2022 Nov 30;612(7940):488–94. doi: 10.1038/s41586-022-05480-9 (PMC9750870; doi:10.1038/s41586-022-05480-9)
Supplement: Supplementary file 1 — This file contains Supplementary Discussion and Supplementary References. [file 41586_2022_5480_MOESM1_ESM.docx]

Supplementary Discussion

Our results show that the pupal social fluid has the molecular characteristics of insect molting fluids, and that it is rich in a variety of proteins and metabolites (Fig. 2). This opens the possibility that the pupal social fluid, when consumed by larvae and adults, has effects on the development, physiology, and behavior of nestmates. GO enrichment analysis of proteins found in the secretion identified functions involved in the regulation of growth and development, including ‘regulation of tube size’ and ‘imaginal disc-derived genitalia morphogenesis’ (Extended Data Fig. 3). Other functions are related to neurological processes involved in development, including ‘motor-neuron axon guidance’, ‘negative chemotaxis’ and ‘synaptic target inhibition’ (Extended Data Fig. 3). While these functions evidently play a role in pupal development, it is reasonable to hypothesize that they might also affect larval development, as well as the physiology and behavior of larvae and adults. Furthermore, similar to mammalian milk, the pupal social fluid contains proteolytic enzymes such as trypsin, chymotrypsin, endopeptidase and lysozyme (Fig. 2b, Extended Data Fig. 2) that might assist in digestion^1^ and protect against pathogens^2^.

Among the metabolites and proteins found in the pupal social fluid (Extended Data Figs. 1 & 3-4), we identified biologically active compounds such as imaginal disc growth factor 4 (Idgf4)^3^ and hormones such as vitellogenin^4^ and histamine^5^. These compounds are involved in different social functions in other insects. For example, Idgf4 is found in the trophallactic fluids of adult ants and honeybees^6,7^. Histamine is a component of the aggregation pheromone in bed bug feces^8^. Vitellogenin, a major egg yolk precursor protein in insects, is involved in the reproductive division of labor in eusocial insects and hormonal dynamics in the worker caste^4,9–11^. We also found three hexamerin proteins in pupal social fluid, and the expression patterns of these proteins correlate with caste in other eusocial insect species^12-14^.

Vitellogenin and hexamerins also have a role as nutrient storage proteins. In holometabolous insects, these proteins accumulate during larval development and serve as an amino acid source during the pupal and pharate adult stages^15^. Therefore, together with other nutrient storage proteins found in the pupal social fluid, such as a yellow/major royal jelly protein (MRJP)^16^, these proteins might participate in the circulation of nutrients in the colony.

Some of the molecules found in the pupal social fluid are major components of honeybee royal jelly. These include MRJP, Idgf4, and apolipophorin^17^. In honeybees, female caste development is nutritionally regulated in that only larvae that are exclusively fed on royal jelly develop into queens^18^. Our findings raise the possibility that the pupal social fluid consumed by ant larvae could play a role in determining their trajectory of caste development.

What young ant larvae feed on is often not clear, and in several species, observations suggest that they do not receive the same diet of solid food as older larvae of the same species^19,20,21^ (Supplementary Video 5). Instead, in species with adult-to-larva trophallaxis such as *S. invicta*, it has been suggested that young larvae receive liquid food from workers via mouth-to-mouth feeding^22^. In other species, young larvae consume trophic or even viable eggs^19,23^. While there is no evidence for adult-to-larva trophallaxis in *O. biroi*, it is possible that young larvae in this species consume eggs, which are present during the colony cycle when larvae hatch^24^. How exactly pupal secretions, eggs, adult trophallactic fluids, and external food sources contribute to early larval nutrition across the ant phylogeny should be a focus of future research.

During the reproductive phase when colonies contain pupae (Fig. 3a), *O. biroi* adults do not leave the nest to forage and retrieve food. Instead, they pile up the pupae and remain in prolonged physical contact with them. We show that adults consume the pupal social fluid (Fig. 1f) and place newly hatched larvae on secreting pupae to provide them access to the pupal fluid as well (Fig. 3c-h). In other ant species, where different developmental stages overlap more broadly, nurses also remain in the nest to take care of pupae and larvae, and our cursory observations suggest that they also place young larvae on pupae (Supplementary Video 5). It is therefore possible that the pupal social fluid modulates nursing behavior in the adults.

The metabolite inventory of the pupal social fluid revealed molecules with known neuroactive functions, including neurotransmitters such as gamma-aminobutyric acid (GABA), glycine, taurine, beta-alanine, glutamic acid, and aspartic acid (Extended Data Figs. 3 & 4). These neurotransmitters are also present in larva-derived trophallactic fluid in hornets, and feeding an artificial blend of these compounds to hornet workers modulates their behavior by decreasing aggression^25^. Other neuroactive compounds identified in the pupal social fluid include N-acetylcysteine, which has neuroprotective functions in rat pups^26^, kynurenic acid, which regulates food-dependent behavioral plasticity in *C. elegans*^27^, adenosine, which can increase [appetite](https://en.wikipedia.org/wiki/Appetite) and food consumption^28^, as well as histamine, which is involved in sleep-wake regulation and modulates different behaviors and homeostatic functions^29^. In principle, these molecules can modulate nestmate neurophysiology either through direct interactions with receptors in the gastrointestinal tract, or following absorption/passive diffusion through the gut wall into the hemolymph.

Two recent studies have shown that the behavior of adult ants can indeed be modulated via consumed secretions of social partners. The secretions of symbiotic caterpillars of lycaenid butterflies, for example, alter dopamine levels in attending ants to reduce their locomotor activity and increase partner fidelity^30^. Similarly, some aphids increase aggressiveness of their mutualistic ants via dopamine in honeydew^31^. Our study suggests that the proclivity to consume liquid secretions from pupae is an ancient evolutionary innovation in ants that precedes the evolution of mouth-to-mouth trophallaxis. This behavior could then have been co-opted in the evolution of symbiotic interactions with different types of other organisms, as has been suggested for trophallaxis by W. M. Wheeler^32^.

**Supplementary References**

1. N. Khaldi, V. Vijayakumar, D. C. Dallas, A. Guerrero, S. Wickramasinghe, J. T. Smilowitz, J. F. Medrano, C. B. Lebrilla, D. C. Shields, J. B. German, Predicting the important enzymes in human breast milk digestion. *J. Agric. Food Chem.* **62**, 7225–7232 (2014).

2. E. E. Ella, A. A. Ahmad, W. N. Ogala, A. Musa, Studies on the interaction between IgA, lactoferrin and lysozyme in the breastmilk of lactating women with sick and healthy babies. *J. Infect. Dis. Immun.* **3**, 24–29 (2011).

3. K. Kawamura, T. Shibata, O. Saget, D. Peel, P. J. Bryant, A new family of growth factors produced by the fat body and active on *Drosophila* imaginal disc cells. *Development*. **126**, 211–219 (1999).

4. A. Hefetz, C. M. Grozinger, Hormonal Regulation of Behavioral and Phenotypic Plasticity in Bumblebees in *Hormones, Brain and Behavior: Third Edition,* D. W. Pfaff, M. Joëls, Eds. (Academic Press, Cambridge, MA, 2017), vol. 2, pp. 453–464.

5. A. A. Al-Badr, H. I. El-Subbagh, Histamine in *Analytical Profiles of Drug Substances and Excipients*, K. Florey, Ed. (Academic Press, Cambridge, MA, 2001), vol. 27, pp. 159–264.

6. A. C. LeBoeuf, P. Waridel, C. S. Brent, A. N. Gonçalves, L. Menin, D. Ortiz, O. Riba-Grognuz, A. Koto, Z. G. Soares, E. Privman, E. A. Miska, R. Benton, L. Keller, Oral transfer of chemical cues, growth proteins and hormones in social insects. *Elife*. **5**, e20375 (2016).

7. T. Fujita, H. Kozuka-Hata, Y. Uno, K. Nishikori, M. Morioka, M. Oyama, T. Kubo, Functional analysis of the honeybee (*Apis mellifera* L.) salivary system using proteomics. *Biochem. Biophys. Res. Commun.* **397**, 740–744 (2010).

8. R. Gries, R. Britton, M. Holmes, H. Zhai, J. Draper, G. Gries, Bed bug aggregation pheromone finally identified. *Angew. Chemie*. **127**, 1151–1154 (2015).

9. K. R. Guidugli, A. M. Nascimento, G. V. Amdam, A. R. Barchuk, S. Omholt, Z. L. P. Simões, K. Hartfelder, Vitellogenin regulates hormonal dynamics in the worker caste of a eusocial insect. *FEBS Lett.* **579**, 4961–4965 (2005).

10. R. Libbrecht, M. Corona, F. Wende, D. O. Azevedo, J. E. Serrão, L. Keller, Interplay between insulin signaling, juvenile hormone, and vitellogenin regulates maternal effects on polyphenism in ants. *Proc. Natl. Acad. Sci. U. S. A.* **110**, 11050–11055 (2013).

11. C. M. Nelson, K. E. Ihle, M. K. Fondrk, R. E. Page, G. V. Amdam, The gene vitellogenin has multiple coordinating effects on social organization. *PLoS Biol.* **5**, 0673–0677 (2007).

12. X. Zhou, M. R. Tarver, M. E. Scharf, Hexamerin-based regulation of juvenile hormone-dependent gene expression underlies phenotypic plasticity in a social insect. *Development*. **134**, 601–610 (2007).

13. J. M. Jandt, A. L. Toth, Physiological and genomic mechanisms of social organization in wasps (family: Vespidae) in *Advances in Insect Physiology*, Z.Amro, F.K. Clement, Eds. (Academic Press, Cambridge, MA, 2015), vol. 48, pp. 95–130.

14. C. Hawkings, T. L. Calkins, P. V. Pietrantonio, C. Tamborindeguy, Caste-based differential transcriptional expression of hexamerins in response to a juvenile hormone analog in the red imported fire ant (*Solenopsis invicta*). *PLoS One*. **14**, e0216800 (2019).

15. W. H. Telfer, J. G. Kunkel, The function and evolution of insect storage hexamers. *Annu. Rev. Entomol.* **36**, 205–228 (1991).

16. M. D. Drapeau, S. Albert, R. Kucharski, C. Prusko, R. Maleszka, Evolution of the yellow/major royal jelly protein family and the emergence of social behavior in honey bees. *Genome Res.* **16**, 1385–1394 (2006).

17. T. Fujita, H. Kozuka-Hata, H. Ao-Kondo, T. Kunieda, M. Oyama, T. Kubo, Proteomic analysis of the royal jelly and characterization of the functions of its derivation glands in the honeybee. *J. Proteome Res.* **12**, 404–411 (2013).

18. W. von Rhein, Über die Entstehung des weiblichen Dimorphismus im Bienenstaate. *Wilhelm Roux. Arch. Entwickl. Mech. Org.* **129**, 601–665 (1933).

19. B. Hölldobler, E. O. Wilson, *The Ants* (Harvard University Press, Cambridge, MA, 1990).

20. W. M. Wheeler, A study of some ant larvæ , with a consideration of the origin and meaning of the social habit among insects. *Proc. Am. Philos. Soc.* **57**, 293–343 (1918).

21. W. R. Tschinkel, *The Fire Ants* (Harvard University Press, Cambridge, MA, 2006).

22. R. S. Petralia, S. B. Vinson, Feeding in the larvae of the imported fire ant, *Solenopsis invicta*: Behavior and morphological adaptations. *Ann. Entomol. Soc. Am.* **71**, 643–648 (1978).

23. B. J. Crespi, Cannibalism and trophic eggs in subsocial and eusocial insect in *Cannibalism: ecology and evolution among diverse taxa*, M. A. Elgar, B. J. Crespi, Eds. (Oxford University Press, Oxford, 1992), pp. 176–213.

24. F. Ravary, P. Jaisson, The reproductive cycle of thelytokous colonies of *Cerapachys biroi* Forel (Formicidae, Cerapachyinae). *Insectes Soc.* **49**, 114–119 (2002).

25. A. Takashi, T. Yoshiya, M. Hiromitsu, K. Yasuko Y., Comparative study of the composition of hornet larval saliva, its effect on behaviour and role of trophallaxis. *Comp. Biochem. Physiol. Part C, Comp.* **99**, 79–84 (1991).

26. L. K. Bernhardt, K. L. Bairy, S. Madhyastha, Neuroprotective role of N-acetylcysteine against learning deficits and altered brain neurotransmitters in rat pups subjected to prenatal stress. *Brain Sci.* **8**, 120 (2018).

27. G. A. Lemieux, K. A. Cunningham, L. Lin, F. Mayer, Z. Werb, K. Ashrafi, Kynurenic acid is a nutritional cue that enables behavioral plasticity. *Cell*. **160**, 119–131 (2015).

28. L. Wu, J. Meng, Q. Shen, Y. Zhang, S. Pan, Z. Chen, L. Q. Zhu, Y. Lu, Y. Huang, G. Zhang, Caffeine inhibits hypothalamic A 1 R to excite oxytocin neuron and ameliorate dietary obesity in mice. *Nat. Commun.* **8**, 1–15 (2017).

29. M. B. Passani, P. Panula, J. S. Lin, Histamine in the brain. *Front. Syst. Neurosci.* **8,** 64 (2014).

30. M. K. Hojo, N. E. Pierce, K. Tsuji, Lycaenid caterpillar secretions manipulate attendant ant behavior. *Curr. Biol.* **25**, 2260–2264 (2015).

31. T. Kudo, H. Aonuma, E. Hasegawa, A symbiotic aphid selfishly manipulates attending ants via dopamine in honeydew. *Sci. Rep.* **11**, 1–8 (2021).

32. W. M. Wheeler, *Social Life Among the Insects* (Harcourt, Brace and Co. New York, NY, 1923).
