# Supplementary material for: The pupal moulting fluid has evolved social functions in ants
Source: Nature. 2022 Nov 30;612(7940):488–94. doi: 10.1038/s41586-022-05480-9 (PMC9750870; doi:10.1038/s41586-022-05480-9)
Supplement: Supplementary file 2 — Reporting Summary [file 41586_2022_5480_MOESM2_ESM.pdf]

nature portfolio

Corresponding author(s): Daniel Kronauer

Last updated by author(s): Oct 5, 2022

Reporting Summary

Nature Portfolio wishes to improve the reproducibility of the work that we publish. This form provides structure for consistency and transparency in reporting. For further information on Nature Portfolio policies, see our Editorial Policies and the Editorial Policy Checklist.

Statistics

For all statistical analyses, confirm that the following items are present in the figure legend, table legend, main text, or Methods section.

n/a	Confirmed	
☐	☒	The exact sample size (*n*) for each experimental group/condition, given as a discrete number and unit of measurement
☐	☒	A statement on whether measurements were taken from distinct samples or whether the same sample was measured repeatedly
☐	☒	The statistical test(s) used AND whether they are one- or two-sided<br>*Only common tests should be described solely by name; describe more complex techniques in the Methods section.*
☒	☐	A description of all covariates tested
☐	☒	A description of any assumptions or corrections, such as tests of normality and adjustment for multiple comparisons
☐	☒	A full description of the statistical parameters including central tendency (e.g. means) or other basic estimates (e.g. regression coefficient) AND variation (e.g. standard deviation) or associated estimates of uncertainty (e.g. confidence intervals)
☐	☒	For null hypothesis testing, the test statistic (e.g. *F*, *t*, *r*) with confidence intervals, effect sizes, degrees of freedom and *P* value noted<br>*Give P values as exact values whenever suitable.*
☒	☐	For Bayesian analysis, information on the choice of priors and Markov chain Monte Carlo settings
☒	☐	For hierarchical and complex designs, identification of the appropriate level for tests and full reporting of outcomes
☐	☒	Estimates of effect sizes (e.g. Cohen's *d*, Pearson's *r*), indicating how they were calculated

Our web collection on statistics for biologists contains articles on many of the points above.

Software and code

Policy information about availability of computer code

Data collection	VisiView acquisition software v 4.5.0.13 (VisiTech International, UK)<br>Leica Application Suite v 4.12.0 (Leica Microsystems, Switzerland)
Data analysis	MATLAB R2020b (MathWorks)<br>R v 3.6.3<br>Proteome Discoverer v.1.4 (Thermo Scientific)<br>EggNog-Mapper tool (http://eggnog-mapper.embl.de, emapper version 1.0.3-35-g63c274b, EggNogDB version 2)<br>clusterProfiler package (R)<br>Skyline Daily v 20.1 (MacCoss Lab)<br>MetaboAnalyst v 4.0<br>ImageJ/Fiji v 1.52p

For manuscripts utilizing custom algorithms or software that are central to the research but not yet described in published literature, software must be made available to editors and reviewers. We strongly encourage code deposition in a community repository (e.g. GitHub). See the Nature Portfolio guidelines for submitting code & software for further information.

Data

Policy information about availability of data

All manuscripts must include a data availability statement. This statement should provide the following information, where applicable:

- Accession codes, unique identifiers, or web links for publicly available datasets
- A description of any restrictions on data availability
- For clinical datasets or third party data, please ensure that the statement adheres to our policy

The main data supporting the findings of this study are available within the paper and its Supplementary Information. Mass spectrometry proteomics data have been uploaded to the PRIDE database (accession number PXD037344).
Databases used in the study are UniProtKB https://www.uniprot.org/ and HMDB https://hmdb.ca/

Field-specific reporting

Please select the one below that is the best fit for your research. If you are not sure, read the appropriate sections before making your selection.

☒ Life sciences ☐ Behavioural & social sciences ☐ Ecological, evolutionary & environmental sciences

For a reference copy of the document with all sections, see nature.com/documents/nr-reporting-summary-flat.pdf

Life sciences study design

All studies must disclose on these points even when the disclosure is negative.

Sample size	Sample size calculations were not performed for the experiments. Appropriate sample sizes were estimated based on preliminary data that showed strong effects with small variations.
Data exclusions	No data was exluded.
Replication	Replication was done using individuals from different stock colonies at different dates to minimize batch effects. All attempts of replication were successful. All experiments were replicated three times or more, except the metabolomic profiling in Fig. 4b in which each sample contained secretion from 30 individuals that were pooled, as well as the scanning electron microscopy image of a P. pennsylvanica cocoon in Extended Data Fig. 8a.
Randomization	Experimental ants were collected haphazardly from stock colonies and distributed across experimental colonies/setups at random. The order of collection and place in which the colonies/setups were arranged were randomized.
Blinding	Blinding was not applicable in this study due to the descriptive nature of the experiments.

Reporting for specific materials, systems and methods

We require information from authors about some types of materials, experimental systems and methods used in many studies. Here, indicate whether each material, system or method listed is relevant to your study. If you are not sure if a list item applies to your research, read the appropriate section before selecting a response.

Materials & experimental systems

n/a	Involved in the study	
☒	☐	Antibodies
☒	☐	Eukaryotic cell lines
☒	☐	Palaeontology and archaeology
☐	☒	Animals and other organisms
☒	☐	Human research participants
☒	☐	Clinical data
☒	☐	Dual use research of concern

Methods

n/a	Involved in the study	
☒	☐	ChIP-seq
☒	☐	Flow cytometry
☒	☐	MRI-based neuroimaging

Animals and other organisms

Policy information about studies involving animals; ARRIVE guidelines recommended for reporting animal research

Laboratory animals	The study included one to several months old Ooceraea biroi females, as well as females of different developmental stages (larvae and pupae). Only female ants display social behavior, and all worker ants are female. In O. biroi in particular, males are only produced sporadically and do not partake in the social life of the colony. Conducting experiments with males is therefore not relevant in the context of the current study.

Wild animals	*Provide details on animals observed in or captured in the field; report species, sex and age where possible. Describe how animals were caught and transported and what happened to captive animals after the study (if killed, explain why and describe method; if released, say where and when) OR state that the study did not involve wild animals.*
Field-collected samples	Nylanderia flavipes, Tapinoma sessile, Ponera pennsylvanica, Solenopsis invicta, Lasius neoniger and Myrmecocystus mexicanus (no ethics guidelines apply).
Ethics oversight	No ethical approval was required since the animals used in this study are insects.

Note that full information on the approval of the study protocol must also be provided in the manuscript.
